# Biological characterization of *Trypanosoma cruzi* epimastigotes derived from trypomastigotes isolated from Brazilian chagasic patients

**DOI:** 10.1016/j.crmicr.2022.100110

**Published:** 2022-02-09

**Authors:** Isabella Bagni Nakamura, Danilo Ciccone Miguel, Andressa Bruscato, Mariane Barroso Pereira, Dimas Campiolo, Eros Antônio de Almeida, Eduardo de Figueiredo Peloso, Fernanda Ramos Gadelha

**Affiliations:** a Departamento de Bioquímica e Biologia Tecidual, Instituto de Biologia, UNICAMP, Campinas, São Paulo, 13083-862, Brazil; b Departamento de Biologia Animal, Instituto de Biologia, UNICAMP, Campinas, São Paulo, 13083-862, Brazil; c Departamento de Clínica Médica, Faculdade de Ciências Médicas, UNICAMP, Campinas, São Paulo, 13083-894, Brazil; d Departamento de Bioquímica, Instituto de Ciências Biomédicas, UNIFAL, MG, 37130-001, Brazil

**Keywords:** *Trypanosoma cruzi*, Mitochondrial bioenergetics, Chagas disease

## Abstract

Chagas disease (CD), caused by *Trypanosoma cruzi*, occurs in several countries in Latin America and non-endemic countries. Heterogeneity among *T. cruzi* population has been the Achilles’ heel to find a better treatment for CD. In this study, we characterized the biochemical parameters and mitochondrial bioenergetics of epimastigotes differentiated from eight *T. cruzi* isolates (I1-I8) obtained from Brazilian CD patients. Molecular analysis of parasites DTUs grouped all of them as TcII. The profile of the growth curves in axenic cultures was distinct among them, except for I1 and I3 and I2 and I4. Doubling times, growth rates, cell body length, and resistance to benznidazole were also significantly different among them. All the isolates were more glucose-dependent than other *T. cruzi* strains adapted to grow in axenic culture. Mitochondrial bioenergetics analysis showed that each isolate behaved differently regarding oxygen consumption rates in non-permeabilized and in digitonin-permeabilized cells in the presence of a complex II-linked substrate. When complex IV-linked respiratory chain substrate was used to provide electrons to the mitochondrial respiratory chain (MRC), similarity among the isolates was higher. Our findings show that TcII epimastigotes derived from patients’ trypomastigotes displayed their own characteristics *in vitro*, highlighting the intra-TcII diversity, especially regarding the functionality of mitochondrial respiratory complexes II and IV. Understanding *T. cruzi* intraspecific biological features help us to move a step further on our comprehension regarding parasite's survival and adaptability offering clues to improve the development of new therapies for CD.

## Highlights

• *T. cruzi* TcII isolates from chagasic patients have distinct biological parameters.
• Isolates were more glucose-dependent than long-term cultivated Y strain parasites.
• Significant differences were observed on complex II and IV-supported respiration.

## Graphical abstract

**Figure:**
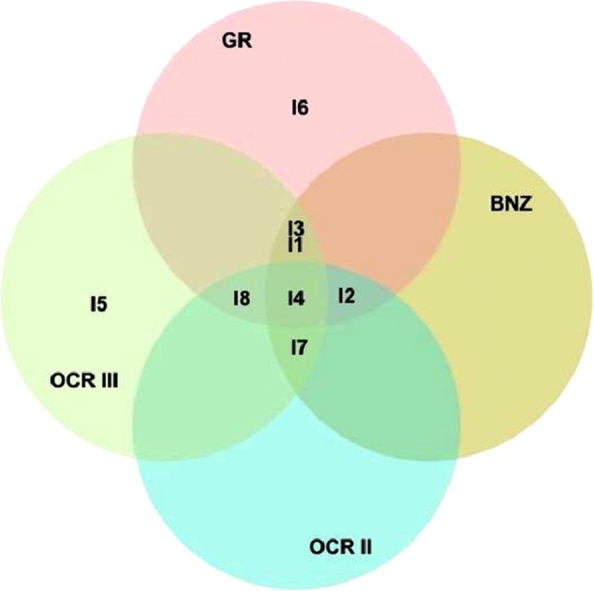

## Abbreviations

***Trypanosoma cruzi* TcII isolates (I1-I8) were distributed by their rates above the average of all the isolates in each parameter, i.e.,**
GR: Growth Rate
BNZ: resistance to benznidazole
OCR II: Oxygen respiratory rates supported by Complex II substrate
OCR IV: Oxygen respiratory rates supported by Complex IV substrate.

## 1 Introduction

Chagas disease (CD), a potentially life-threatening disease, is caused by *Trypanosoma cruzi*, a protozoan of the Trypanosomatidae family. CD affects approximately 6–7 million people worldwide, mostly in Latin America (WHO, 2021). Current treatment is restricted to nitroheterocyclic drugs, nifurtimox, and benznidazole (BNZ) that are effective if given at the onset of the acute phase, but possibly lead to severe side effects (WHO, 2021). Responsiveness to BNZ, the most common drug used, is diverse among *T. cruzi* strains (Zingales, 2018) and the genetic and phenotype heterogeneity of *T. cruzi* population has been pointed out as the Achilles’ heel to the development of more effective treatments.

*T. cruzi* populations have a broad biological diversity being arranged into six major discrete typing units (DTUs) (TcI-TcVI), each one bearing distinct geographical and biological characteristics (Zingales et al., 2012). A seventh group includes bats-isolated parasites (Tcbat), with one human infection case reported (Ramírez et al., 2014). TcI has the widest geographical distribution, and it is the main DTU responsible for CD in Colombia and Venezuela, while TcII, TcV, and TcVI are detected in southern areas of South America. TcIII has a scattered distribution from northeastern Venezuela to Argentina (Zingales et al., 2012). Even though *T. cruzi* population has been distributed among DTUs, sub-DTU variation has been pointed out, especially in TcI (Zingales, 2018).

In the present work, eight *T. cruzi* isolates from Brazilian CD chronic patients were differentiated into epimastigotes and we analyzed their biological parameters, emphasizing growth behavior, and mitochondrial bioenergetics in the presence of different substrates.

## 2 Materials and methods

### 2.1 *T. cruzi* samples

A total of eight isolates (I1-I8) were obtained from chronic CD patients randomly selected from scheduled appointments at the Study Group on CD of the State University of Campinas Clinical Hospital, São Paulo, Brazil. The Institutional Ethics Committee approved this study (CAAE 42839615.0.0000.5404; 22598719.2.0000.5404).

### 2.2 Differentiation and culture establishment of epimastigotes

Blood collected from each patient was added to the LIT medium (LIT supplemented with 20 mg/L hemin, 10% fetal calf serum, and penicillin and streptomycin) and kept at 28 °C (Castellani et al., 1967). Cultures were monitored by optical microscopy every other day to follow the differentiation process. After approximately four weeks, all samples had differentiated epimastigotes, which were grown in the LIT medium at 28 °C. Aliquots of log-phase parasites were stored in liquid nitrogen and fresh aliquots were recovered every two months.

**Growth curves.** For the growth curves, 5 × 10^6^ epimastigotes/mL were cultured in the LIT medium, and on specified days, the number of cells was determined using a Neubauer chamber. From the growth curves, Doubling Time (DT) (Toma et al., 2000) and Growth Rate (GR) (Martinez-Diaz et al., 2001) were calculated. For biochemical assays, experiments were conducted using parasites at the end of the log phase, which were harvested by centrifugation (1000 × *g* at 4 °C), washed, and resuspended in IB medium (5 mM KCl; 80 mM NaCl; 2 mM MgCl_2_; 16.2 mM Na_2_HPO_4_; 3.8 mM NaH_2_PO_4_; 50 mM d-glucose and BSA 0.15% adjusted to pH 7.4).

**Light microscopy.** 2.6 × 10^7^ cells/mL in the early stationary phase were added to polylysine slides and allowed to dry. Then, the Rapid Panoptic LB Laborclin kit was used to stain the cells. The coverslips were added and sealed with Entellan (Merck). The images were captured in an ICC50-HD camera coupled to a Leica DM500 microscope and processed using Leica Application Suite software V. 4.2.0 (Leica Microsystems Co., Germany).

**Benznidazole susceptibility.** EC_50_ was determined by the MTT assay as previously described (Zauli-Nascimento et al., 2010). Briefly, 5.2 × 10^6^ epimastigotes/mL were grown in the presence of different concentrations of BNZ in the LIT medium at 28 °C. When parasites reached the early-stationary phase, 30 μL of 5 mg/mL MTT (3-[4,5-*dimethyl*-2-*thiazolyl*]−2,5-diphenyl-2H-tetrazolium bromide) was added to each well. After incubation for another 3 h, 50 μL of 20% SDS was added to each well. Absorbance was determined in a Cytation 5™ cell imaging multi-mode reader with a reference and test wavelength of 650 and 600 nm, respectively.

**DTU determination.** DNA was extracted by the proteinase K method (Sambrook and Russell, 2001). PCR was employed to genotype *T. cruzi* isolates, as previously described (Lewis et al., 2009).

**Oxygen uptake determination.** O_2_ consumption was monitored in a computer-interfaced Clark-type oxygen electrode (Hansatech® Systems Inc., Norfolk, Eng.) under two conditions: non-permeabilized and permeabilized cells. In the first, 10^8^ cells/mL were resuspended in IB in the absence or presence of 1 μM CCCP, an uncoupler of oxidative phosphorylation. In parallel, 10^8^/mL were incubated in IB medium in the presence of 20 μM digitonin and 5 mM succinate (complex II-linked substrate) or 100 μM TMPD/ascorbate (complex IV-linked substrate). The Respiratory Control Ratio (RCR) was determined by the addition of 400 μM ADP followed by 1 μg/mL oligomycin (Silva et al., 2011).

**Statistical analysis.** Data of experiments represent means ± standard deviations of three independent experiments performed at least in duplicates. One Way Anova, Tukey's multiple comparisons test was employed, where significant differences at *p* < 0.05 were identified by letters "a–h" as compared to I1, I2, I3, I4, I5, I6, I7, and I8, respectively.

## 3 Results and discussion

Patients from whom the parasites were isolated came from different regions of Brazil attended the State University of Campinas Hospital (HC-UNICAMP) being diagnosed with chronic CD. The majority of them did not know how they were infected and each one had a particular profile regarding BNZ treatment and family history. All *T. cruzi* isolates were genotyped as TcII. Corroborating our results, Zingales compiled the results from a survey with 375 CD Brazilian patients (Brenière et al., 2016) and found that TcII had the highest prevalence (66.1%) (Zingales, 2018).

**Fig. 1:**
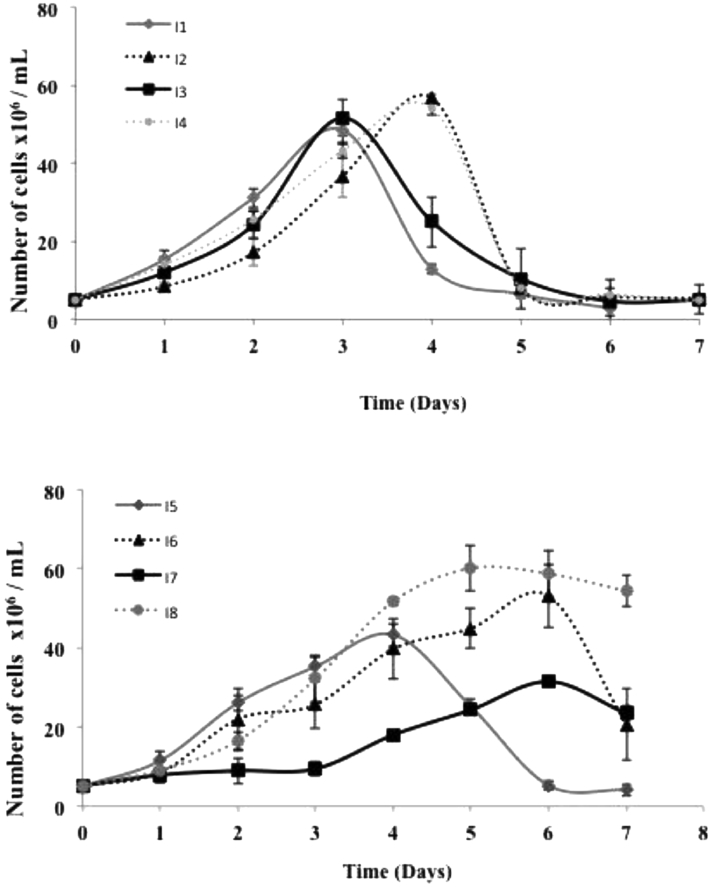
*Trypanosoma cruzi* epimastigotes growth curves. Epimastigotes, derived from trypomastigotes isolated from Brazilian Chagas disease patients (I1–I8), were incubated in the culture medium, and on the days specified, the number of cells was determined using a Neubauer chamber.

Epimastigotes’ growth curves are represented in Fig. 1. Four of the isolates (I1, I3, I4, and I5) did not have a defined lag phase, according to a similar study performed with parasites also isolated from CD chronic patients (Oliveira et al., 2017). Although all isolates were TcII, they displayed distinct growth profiles. For I1 and I3, an early maximum peak of parasite growth was reached on the 3rd day, while for I2, I4, and I5 at the 4th day and I6 and I7 at the 6th day in culture. After reaching this peak, all isolates, but I8, started to die, a feature also reported for other TcII samples from CD patients (Oliveira et al., 2017). I8 had a different profile once a maximum peak was reached on the 5th day and slowly parasites started to die, like I7 (Fig. 1). In the stationary phase, we did not observe trypomastigotes in the cultures. We have previously performed the same experiment with long-term cultivated Y and Tulahuen strains and no sharp decrease in the number of parasites occurred after the stationary phase (Mielniczki-Pereira et al., 2007). *T. cruzi* epimastigotes preferably use glucose as an energy source that is rapidly and partially degraded during the exponential phase (Engel et al., 1987). In the early stationary phase, a metabolic shift from glucose to amino acid consumption is observed with activation of mitochondrial enzymes and an increase in cytochrome content (Henandez et al., 2012). These adaptations, among others, will allow epimastigotes to use amino acids as an energy source. We hypothesized that epimastigotes, which have not been adapted to the culture medium, are less prone to a metabolic shift by using amino acids as an energy source in the stationary phase.

Further analysis of the growth curves enabled us to determine the Doubling Time (DT) and Growth Rate (GR) of the isolates (Table 1). There were significant differences in DT among isolates, especially for I6, I7, and I8. GR analysis also showed significant differences, with I7 having GR values significantly different from all the others. Supporting our results, parasites isolated from patients with cardiac and digestive CD forms showed different growth kinetics, DT, and differentiation in axenic culture (Oliveira et al., 2017; Lauria-Pires et al., 1997).

**Table 1:** Biological parameters of *T. cruzi* epimastigotes.

Isolate	DT (hs)	GR	Cell body length (μm)	EC_50_BNZ (μM)
I1	22.50 ± 0.30^e,f,g,h^	9.22 ± 0.30^b,g,h^	22.77 ± 4.01^e^	3.71 ± 1.57^b^
I2	27.15 ± 0.25^f,g,h^	10.75 ± 0.38^e,g^	24.26 ± 4.13	32.81 ± 5.21 ^b,c,d,e,f,g,h^
I3	21.73 ± 0.63^e,f,g,h^	10.00 ± 0.65^e,g^	20.77 ± 3.26 ^e,h^	4.69 ± 0.32
I4	28.47 ± 0.36^f,g,h^	10.38 ± 0.30^e,g^	24.57 ± 3.98	6.56 ± 1.88^h^
I5	32.17 ± 1.18^f,g^	7.97 ± 0.61^f,g,h^	27.15 ± 3.11 ^f,g^	2.82 ± 0.76
I6	48.08 ± 3.89 ^g,h^	10.02 ± 1.60 ^g^	21.54 ± 3.76 ^f^	2.82 ± 0.82
I7	71.27 ± 10.14^h^	6.27 ± 0.38^h^	22.37 ± 3.76	4.94 ± 0.50
I8	39.23 ± 3.08	11.68 ± 1.26	25.93 ± 4.28	1.46 ± 0.41

**Fig. 2:**
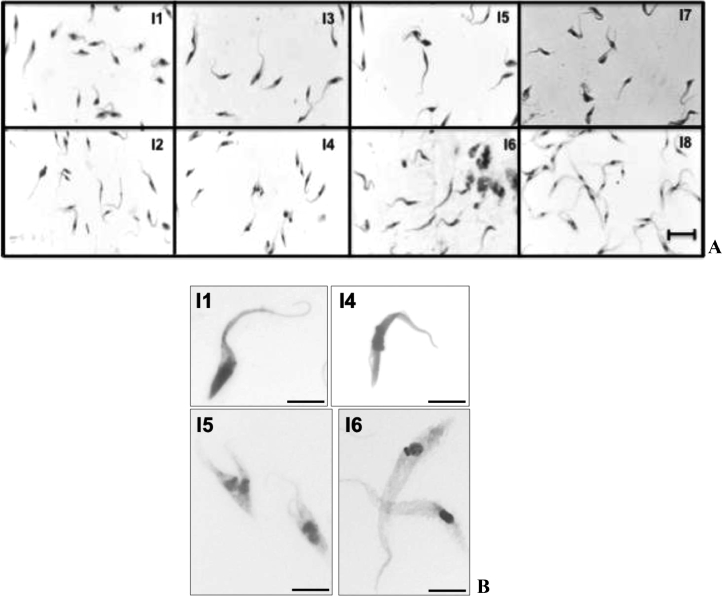
Optical microscopy of *Trypanosoma cruzi* thin smears stained with the panoptic kit. A. Isolates I1-I8 Scale bar: 20μm. B. Representatives of I1, I4, I5, and I6 were zoomed to highlight their unique features in cell body and flagella. Scale bar: 5μm.

Doubling Time (DT) and Growth Rate (GR) were calculated from Fig. 1. From Fig. 2 cell body length was determined (*n* = 5) and for EC_50_ determination early-stationary phase epimastigotes were incubated in the presence of increasing concentrations of BNZ (0–50 μM) and viability was determined by the MTT assay as described in the ‘Material and methods’ section. Statistical analysis: One Way Anova, Tukey's multiple comparisons test, where significant differences at *p* < 0.05 were identified by letters a–h as compared to I1, I2, I3, I4, I5, I6, I7 and I8, respectively.

Morphometric analyses of the culture smears showed that there were also significant differences in cell body length (Fig. 2A, Table 1). I6 parasites showed more slender and elongated cell bodies, and I4 parasites had shorter flagella than I1 and I5 parasites (Fig. 2B).

Next, we investigated the isolates’ resistance to BNZ (Table 1). EC_50_ average for all isolates, but I2 was 3.86 ± 1.69 μM and only I2 displayed a higher EC_50_ (32.81 ± 5.21 μM). Revollo and collaborators found an EC_50_ average value of 1.52 ± 0.54 μM for TcII epimastigotes (Revollo et al., 2019). Heterogeneity among EC_50_ values for BNZ was also found among TcI strains (Martínez et al., 2013).

The intraspecific population variability concerning gene constitution, the immune response of the host, pathogenicity, virulence, and morphology may be associated with the adaptation and survival of *T. cruzi* in its different hosts (Villa et al., 2013), and perhaps as an adaptability response to nutrients contained in the culture medium during the transition from trypomastigote to the epimastigote stage. Strains differences have been observed at the level of the enzymes of the pentose phosphate pathway (Mielniczki-Pereira et al., 2007), tryparedoxin peroxidases, superoxide dismutases (Peloso et al., 2012), trans-sialidase (Burgos et al., 2013), and in oxidative metabolism (Silva et al., 2011). Perhaps these alterations in critical enzymes for *T. cruzi* play a role in the modulation of parasites' adaptability, survival, and pathogenicity.

**Fig. 3:**
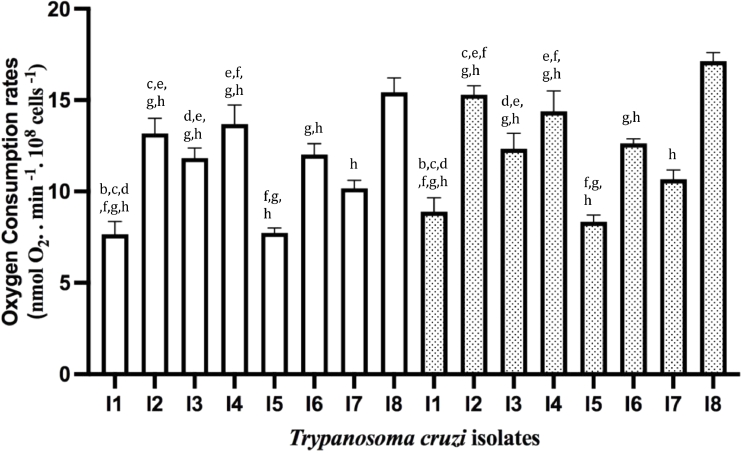
Oxygen consumption in non-permeabilized *Trypanosoma cruzi* epimastigotes. Oxygen consumption rates were determined in the absence (white bars) or presence of CCCP (dotted bars). Statistical analysis: One way anova, Tukey's multiple comparisons test, where significant differences at *p* < 0.05 were identified by letters a-h as compared to I1, I2, I3, I4, I5, I6, I7, and I8, respectively.

We then investigated the mitochondrial bioenergetics of the isolates. Firstly, we determined the oxygen consumption rates (OCR) in non-permeabilized cells, i.e., in the physiological resting respiration (ROUTINE respiration) (Pesta and Gnaiger, 2012) in the absence or presence of CCCP (Fig. 3). The addition of CCCP enabled the determination of mitochondrial respiratory chain's maximal capacity via chemical dissipation of the mitochondrial membrane potential. Relevant differences were found among isolates, with I7 having OCRs values distinct from the others. Surprisingly, an increase in OCR by CCCP was only significant for I2 and I8, suggesting that the mitochondrial respiratory chain was already at its maximum velocity for I1, I3-I7.

**Fig. 4:**
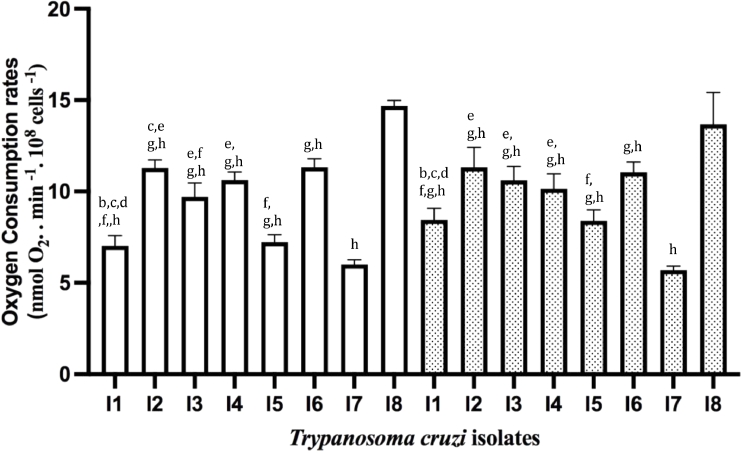
*Trypanosoma cruzi* oxygen consumption rates in the presence of complex II of the mitochondrial respiratory chain. OCR in digitonin-permeabilized cells was determined in the presence of succinate in the absence (white bars) or presence of CCCP (dotted bars). Statistical analysis: One way anova, Tukey's multiple comparisons test, where significant differences at *p* < 0.05 were identified by letters a–h as compared to I1, I2, I3, I4, I5, I6, I7, and I8, respectively.

**Fig. 5:**
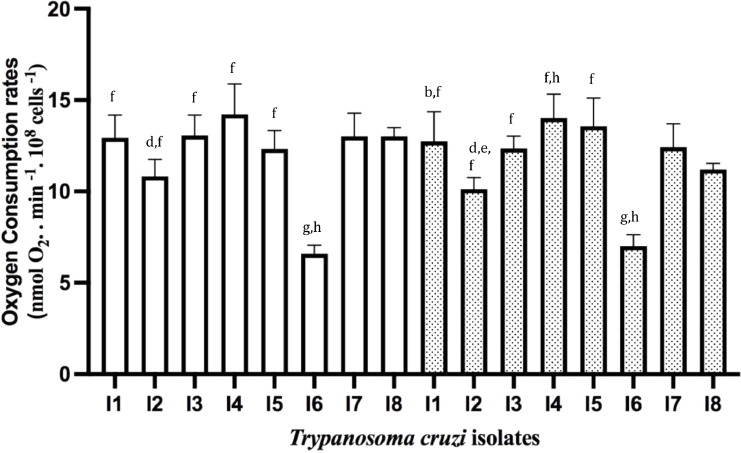
*Trypanosoma cruzi* oxygen consumption rates in the presence of complex IV of the mitochondrial respiratory chain. OCR in digitonin-permeabilized cells was determined in the presence of TMPD/Ascorbate in the absence (white bars) or presence of CCCP (dotted bars). Statistical analysis: One way anova, Tukey's multiple comparisons test, where significant differences at *p* < 0.05 were identified by letters a-h as compared to I1, I2, I3, I4, I5, I6, I7, and I8, respectively.

One of the relevant parameters to determine mitochondrial function is the RCR, which reveals ATP production rate. RCR is the result of State 3 (upon addition of ADP) / State 4 (upon addition of oligomycin, an inhibitor of F_o_F_1_ATP synthase). The addition of ADP allows the determination of mitochondria' maximal capacity to utilize O_2_ since substrate oxidation is coupled to ATP synthesis. We used succinate, a substrate for Complex II, and TMPD/Ascorbate for Complex IV to address the functionality of different mitochondrial respiratory chain complexes. When OCR was determined using a buffer that mimics the intracellular environment as described in (Silva et al., 2011), no stimulation was observed upon ADP addition. Since we have successfully used this buffer with other long-term cultivated *T. cruzi* strains (Silva et al., 2011), we speculated, also from our observation from the growth curves (Fig. 1), whether these isolates could have a higher dependence on glucose (Mielniczki-Pereira et al., 2007). Using a glucose-containing medium (IB medium) to determine the OCRs, an increase in OCR upon ADP addition was observed for both substrates (Fig. 4, Fig. 5). Comparing the results obtained with permeabilized (Figs. 4 and 5) and intact cells (Fig. 3), ROUTINE OCR was lower in the former due to endogenous ADP dilution upon plasma membrane permeabilization.

In the presence of succinate (Fig. 4), significant differences were observed among the isolates, where I7 and I8 had OCRs significantly different from all the others. In the presence of CCCP, only in I1 and I5, OCRs were stimulated. It is intriguing that for some of the isolates, OCRs were stimulated in the presence of CCCP, while for others they were not. The lack of CCCP stimulation is unusual for *T. cruzi* strains, as the lack of ADP stimulation in a medium without glucose. We hypothesized that these features could be inherent to freshly transformed epimastigotes obtained from CD patients.

As expected, OCRs were higher in the presence of TMPD/Ascorbate (Fig. 5) that donated electrons to cytochrome *c*, which then transferred them to complex IV while ascorbate reduced TMPD. Comparing the OCRs obtained under this condition a higher similarity was observed in relation to the OCRs determined in the presence of succinate.

RCR values were very similar among the isolates (Table 2) allowing us to infer that coupling of the mitochondrial respiratory chain and ADP phosphorylation leading to ATP production is equivalent in all TcII isolates studied.

**Table 2:** Respiratory control ratios (State 3 / State 4).

Isolates	Respiratory control ratio	
	Succinate	TMPD/Asc
I1	1.05 ± 0.02^c,g^	1.13 ± 0.02
I2	1.11 ± 0.06	1.15 ± 0.02
I3	1.12 ± 0.02	1.11 ± 0.02^h^
I4	1.08 ± 0.00	1.12 ± 0.09
I5	1.07 ± 0.02 ^g^	1.12 ± 0.05
I6	1.07 ± 0.02 ^g^	1.06 ± 0.01^h^
I7	1.15 ± 0.05	1.15 ± 0.01
I8	1.10 ± 0.04	1.22 ± 0.04

Upon determining the O_2_ consumption rates (Figs. 4 and 5), RCR was calculated as the STATE 3/ STATE 4. Statistical analysis: One Way Anova, Tukey's multiple comparisons test, where significant differences at *p* < 0.05 were identified by letters a-h as compared to I1, I2, I3, I4, I5, I6, I7 and I8, respectively.

In the present study, the biochemical characteristics evaluated showed heterogeneity between *T. cruzi* TcII epimastigotes differentiated from chronic CD patients’ trypomastigotes. Our data point to the need to explore the biochemical aspects of these isolates' infectious forms in the future to compare with the data herein reported for extracellular replicative forms.

## Data availability

The datasets generated during and/or analyzed during the current study are available from the corresponding author on reasonable request.

## Author statement

IBN, AB, MBP and DC carried out the experiments. FRG and EFP conceived the original idea. FRG wrote the manuscript with support from DCM, AEA and EFP. FRG supervised the project. DCM provided a critical revision of the *ms*. Both DCM and FRG contributed to the final version of the *ms*.

## Declaration of Competing Interest

The authors declare that they have no known competing financial interests or personal relationships that could have appeared to influence the work reported in this paper.

## References

[bib0001] Brenière S.F., Waleckx E., Barnabé C. (2016). Over six thousand *Trypanosoma cruzi* strains classified into discrete typing units (DTUs): attempt at an inventory. PLoS Negl. Trop. Dis..

[bib0002] Burgos J.M., Risso M.G., Brenière S.F. (2013). Differential distribution of genes encoding the virulence factor trans-sialidase along *Trypanosoma cruzi* discrete typing units. PLoS ONE.

[bib0003] Castellani O., Ribeiro L.V., Fernandes J.F. (1967). Differentiation of *Trypanosoma cruzi* in culture. J. Protozool.

[bib0004] Engel J.C., Cazzulo B.M., Stoppani A.O., Cannata J.J., Cazzulo J.J. (1987). Aerobic glucose fermentation by *Trypanosoma cruzi* axenic culture amastigote-like forms during growth and differentiation to epimastigotes. Mol. Biochem. Parasitol..

[bib0005] Hernández, R., Cevallos, A.M., Nepomuceno-Mejía, T., López-Villaseñor I., 2012. Stationary phase in *Trypanosoma cruzi* epimastigotes as a preadaptive stage for metacyclogenesis. Parasitol. Res. (2012) 111:509–514. DOI 10.1007/s00436-012-2974-y. 10.1007/s00436-012-2974-y 22648053

[bib0006] Lauria-Pires L., Santana J.M., Tavares F.S. (1997). Diversity of *Trypanosoma cruzi* stocks and clones derived from Chagas disease patients: I-behavioral characterization in vitro. Rev. Soc. Bras Med. Trop..

[bib0007] Lewis M.D., Ma J., Yeo M. (2009). Genotyping of *Trypanosoma cruzi*: systematic selection of assays allowing rapid and accurate discrimination of all known lineages. Am. J. Trop. Med. Hyg.

[bib0008] Martínez I., Nogueda B., Martínez-Hernández F. (2013). Microsatellite and mini-exon analysis of Mexican human DTU I *Trypanosoma cruzi* strains and their susceptibility to nifurtimox and benznidazole. Vector-Borne Zoonotic Dis..

[bib0009] Martinez-Diaz R., Escario J.A., Nogal-Ruiz J. (2001). Biological characterization of *Trypanosoma cruzi* strains. Mem. Inst. Oswaldo Cruz.

[bib0010] Mielniczki-Pereira A.A., Chiavegatto C.M., López J.A. (2007). *Trypanosoma cruzi* strains, Tulahuen 2 and Y, besides the difference in resistance to oxidative stress, display differential glucose-6-phosphate and 6-phosphogluconate dehydrogenases activities. Acta Trop..

[bib0011] Oliveira M.T., Branquinho R.T., Alessio G.D. (2017). TcI, TcII and TcVI *Trypanosoma cruzi* samples from Chagas disease patients with distinct clinical forms and critical analysis of in vitro and in vivo behavior, response to treatment and infection evolution in murine model. Acta Trop..

[bib0012] Peloso E.F., Gonçalves C.C., Silva T.M. (2012). Tryparedoxin peroxidases and superoxide dismutases expression as well as ROS release are related to *Trypanosoma cruzi* epimastigotes growth phases. Arch. Biochem. Biophys..

[bib0013] Pesta D., Gnaiger E. (2012). High-resolution respirometry: OXPHOS protocols for human cells and permeabilized fibers from small biopsies of human muscle. Methods Mol. Biol..

[bib0014] Ramírez J.D., Hernández C., Montilla M. (2014). First report of human *Trypanosoma cruzi* infection attributed to TcBat genotype. Zoonoses Public Health.

[bib0015] Revollo S., Oury B., Vela A. (2019). In vitro benznidazole and nifurtimox susceptibility profile of *Trypanosoma cruzi* strains belonging to discrete typing units TcI. TcII and TcV.

[bib0016] Sambrook, J., Russell, D.W., 2001. Molecular cloning: a laboratory manual. 3rd ed. Plainsville, NY: Cold Spring Harbor Laboratory Press.

[bib0017] Silva T.M., Peloso E.F., Vitor S.C. (2011). O_2_ consumption rates along the growth curve: new insights into *Trypanosoma cruzi* mitochondrial respiratory chain. J. Bioenerg. Biomembr..

[bib0018] Toma H.K., Cerávolo I., Guerra H. (2000). *Trypanosoma cruzi*: parasitemia produced in mice does not seem to be related to in vitro parasite-cell interaction. Int. J. Para-sitol..

[bib0019] Villa L.M., Guhl F., Zabala D. (2013). The identification of two *Trypanosoma cruzi* I genotypes from domestic and sylvatic transmission cycles in Colombia based on a single polymerase chain reaction amplification of the spliced-leader intergenic region. Mem. Inst. Oswaldo Cruz.

[bib0020] WHO, 2021 https://www.who.int/en/news-room/fact-sheets/detail/chagas-disease-(american-trypanosomiasis).

[bib0021] Zauli-Nascimento R., Miguel D.C., Yojoyama-Yasunaka J.K.U. (2010). In vitro sensitivity of *Leishmania (Viannia) Braziliensis* and *Leishmania (Leishmania) amazonensis* Brazilian isolates to meglumine antimoniate and amphotericin B. Trop. Med. Int. Health.

[bib0022] Zingales B., Miles M.A., Campbell D.A. (2012). The revised *Trypanosoma cruzi* subspecific nomenclature: rationale, epidemiological relevance and research applications. Infect. Genet. Evol..

[bib0023] Zingales B. (2018). *Trypanosoma cruzi* genetic diversity: something new for something known about Chagas disease manifestations, serodiagnosis and drug sensitivity. Acta Trop.

